# Flavonoids from *Aurantii Fructus Immaturus* and *Aurantii Fructus*: promising phytomedicines for the treatment of liver diseases

**DOI:** 10.1186/s13020-020-00371-5

**Published:** 2020-08-26

**Authors:** Jianzhi Wu, Guangrui Huang, Yajing Li, Xiaojiaoyang Li

**Affiliations:** School of Life Sciences, Beijing University of Chinese Medicine, 11 Bei San Huan Dong Lu, Beijing, 100029 China

**Keywords:** *Aurantii Fructus Immaturus*, *Aurantii Fructus*, Liver diseases, Flavonoids, Hesperidin, Naringenin

## Abstract

**Background:**

Liver diseases and related complications are major sources of morbidity and mortality, which places a huge financial burden on patients and lead to nonnegligible social problems. Therefore, the discovery of novel therapeutic drugs for the treatment of liver diseases is urgently required. *Aurantii Fructus Immaturus* (AFI) and *Aurantii Fructus* (AF) are frequently used herbal medicines in traditional Chinese medicine (TCM) formulas for the treatment of diverse ailments. A variety of bioactive ingredients have been isolated and identified from AFI and AF, including alkaloids, flavonoids, coumarins and volatile oils.

**Main body:**

Emerging evidence suggests that flavonoids, especially hesperidin (HD), naringenin (NIN), nobiletin (NOB), naringin (NRG), tangeretin (TN), hesperetin (HT) and eriodictyol (ED) are major representative bioactive ingredients that alleviate diseases through multi-targeting mechanisms, including anti-oxidative stress, anti-cytotoxicity, anti-inflammation, anti-fibrosis and anti-tumor mechanisms. In the current review, we summarize the recent progress in the research of hepatoprotective effects of HD, NIN, NOB, NRG, TN, HT and ED and highlight the potential underlying molecular mechanisms. We also point out the limitations of the current studies and shed light on further in-depth pharmacological and pharmacokinetic studies of these bioactive flavonoids.

**Conclusion:**

This review outlines the recent advances in the literature and highlights the potential of these flavonoids isolated from AFI and AF as therapeutic agents for the treatment of liver diseases. Further pharmacological studies will accelerate the development of natural products in AFI and AF and their derivatives as medicines with tantalizing prospects in the clinical application.

## Background

Liver diseases and complications are major sources of morbidity and mortality worldwide and have become a significant economic burden and nonnegligible public health issues. Liver diseases include cholestasis, viral and non-viral hepatitis, fatty liver diseases, drug-induced liver injury, liver fibrosis, and devastating end-stage liver disorders, including cirrhosis, hepatocellular carcinoma (HCC), and cholangiocyte carcinoma (CCA) [1, 2]. The poor understanding of the mechanisms of pathogenesis and limited treatment targets and strategies have brought great challenges to the clinical management of liver diseases. At present, most of the therapeutic options for chronic liver diseases are strong oxidant scavengers, which usually have off-target or adverse effects. Once a patient has progressed to irreversible end-stage liver diseases, chemotherapy and liver transplantation remain the only treatment options. Therefore, effective drugs are urgently needed. Traditional Chinese medicine (TCM) has been practiced in China and other eastern Asian countries for more than 2500 years to treat various diseases [3]. Emerging evidence has demonstrated that several traditional Chinese prescriptions exert promising therapeutic effects on many chronic liver diseases [4, 5]. Thus, the active ingredients from these hepatoprotective herbal medicines may become a major source of novel drug discovery for the treatment of liver diseases.

*Aurantii Fructus Immaturus* (AFI) and *Aurantii Fructus* (AF) are both the fruits of rutaceae plant and have been widely used in numerous herbal formulas. Both AFI and AF were first recorded in *Shen Nong Ben Cao Jing*, the earliest monograph concerning TCM [6]. AFI, which are collected from May to June, are the dried immature fruits of *Citrus sinensis Osbeck* (commonly known as sweet oranges), *Citrus aurantium L*. (commonly known as bitter oranges) and their cultivars, such as *Citrus junos*, *Citrus japonica*, and *Citrus bergamia*, while AF, which are harvested in July, are the dried mature fruits of *Citrus aurantium L*. and its cultivars, such as *Citrus limon* and *Citrus aurantifolia* [7]. AFI and AF are mainly produced in the provinces of Hunan, Jiangxi, Sichuan, Chongqing, and Fujian in China, as well as other countries, such as Japan and South Korea [8]. Currently, many TCM patented medicines containing AFI and AF in their formulations, such as Sini Powder, Fucong Granule, Xiaopi Pills and Bran-processed Fructus aurantii Granule, are used for the treatment of fatty liver disease, hepatitis, functional dyspepsia, peptic ulcer and chronic atrophic gastritis [9, 10]. It is noteworthy that AFI and AF are recorded as distinct medicinal materials in the Chinese Pharmacopoeia, not only because AFI and AF are harvested at different stages of fruit growth but also due to their different pharmacological effects and unique clinical applications [11]. AFI can be used alone to eliminate phlegm, treat cancer, cardiovascular diseases and gouty arthritis [12, 13]. AFI is also used in combination with other Chinese herbs including *Paeonia lactiflora*, *Glycyrrhiza uralensis* and *Angelica sinensis* for improved therapeutic effects [14]. Compared to AFI, AF is often employed to treat and prevent acute lung injury, inflammation, obesity, and gastrointestinal dysfunctions [15, 16]. In addition, it has been documented that AFI and AF exhibit hepatoprotective effects including preventing oxidative stress, improving liver lipid metabolism and ameliorating fatty liver injury [16, 17]. Recently, natural compounds in AFI and AF have attracted great attention for their potential bioactivities in the treatment of chronic liver diseases.

## Flavonoids in *Aurantii Fructus Immaturus* and *Aurantii Fructus*

**Fig. 1:**
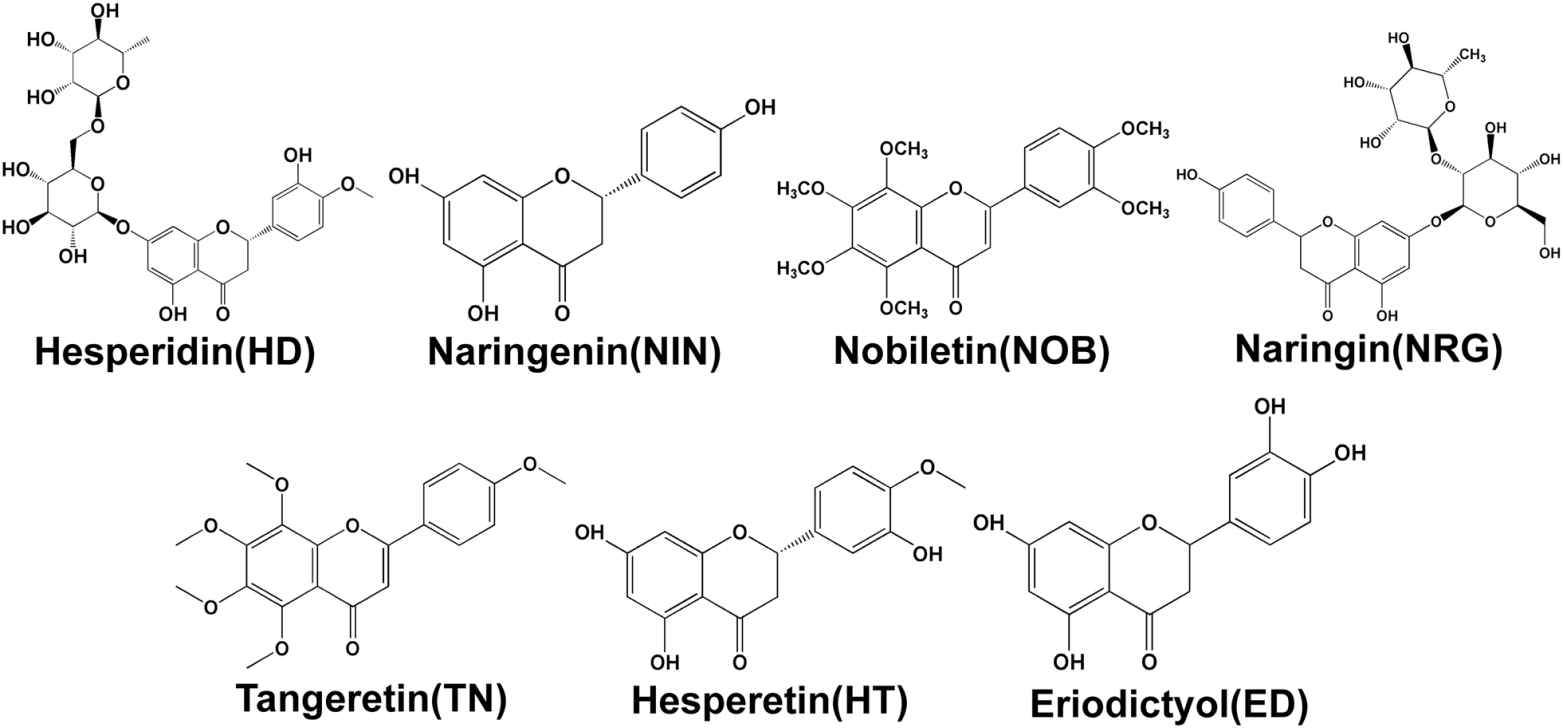
Chemical structures of hesperidin (HD), naringenin (NIN), nobiletin (NOB), naringin (NRG), tangeretin (TN), hesperetin (HT) and eriodictyol (ED)

Thus far, a number of bioactive ingredients from AFI and AF have been isolated and identified, including flavonoids, alkaloids, coumarins and volatile oils [18]. Alkaloids, coumarins and volatile oils are commonly used as food supplements for weight loss or as anti-microbial, anti-fungal and anti-cancer agents, while flavonoids have a variety of medicinal benefits including anti-depressant, anti-oxidant, anti-viral, anti-inflammatory and anti-cancer activities [19–21]. Recently, several flavonoids, such as hesperidin (HD), naringenin (NIN), nobiletin (NOB), naringin (NRG), tangeretin (TN), hesperetin (HT) and eriodictyol (ED) were identified and the amount of the seven flavonoids in AFI were 3.05–212.72, 0.04–1.19, 0.39–30.92, 21.91–169.53, 0.20–29.99, 0.06–0.62, 0.0007–0.29 (mg/g) respectively, while the contents of them in AF were 0.32–30.33, 0.12–2.28, 0.11–35.49, 17.32–75.50, 0.03–5.85, 0.10–2.22, 0.0011–0.03 (mg/g) respectively [6, 22–25]. It should also be noted that the amount of these ingredients in AFI and AF vary depending on the harvesting time and geographical distribution. These flavonoids are believed to be major bioactive ingredients and have been reported to have hepatoprotective effects [26]. The chemical structures of major flavonoids in AFI and AF are shown in Fig. 1. Numerous studies have reported that HD, NIN and NOB are the most potent hepatoprotective ingredients against liver injury induced by clinical ischemia–reperfusion (IR), paraquat (PQ), carrageenan, cyclophosphamide (CYP), carbon tetrachloride (CCl_4_) and other stimulants [27–30]. Recently, several studies have revealed that NRG, TN, HT and ED also contribute to the pharmacological effects of AFI in the treatment of liver diseases.

In this review, we summarize the pharmacological effects of bioactive flavonoids in AFI and AF, including HD, NIN, NOB, NRG, TN, HT and ED, and provide insights into their mechanisms of action and specific targets. In addition, we highlight the prospect of individual products in AFI and AF as therapeutic drugs for the clinical treatment of liver diseases.

## Methods

This review was based on searches of electronic database, including PubMed, Google Scholar, Web of Science and CNKI, using the combination of “*Aurantii Fructus Immaturus*”, “*Aurantii Fructus*”, “Zhi Shi”, “Zhi Qiao”, “hesperidin”, “naringenin”, “nobiletin”, “naringin”, “tangeretin”, “hesperetin” and “eriodictyol” with “liver” as keywords. A total of 161 publications from 2010 to 2020 were retrieved and 116 papers were summarized in this review. Articles regarding the agriculture and basic phytology or secondary metabolite biosynthesis were excluded. Several other articles and reviews were also included to provide essential information and background.

## Pharmacological effects of flavonoids from AFI and AF in liver injury

### Hesperidin

Hesperidin (HD), also named as hesperidoside, has the B ring with OCH_3_ and OH substituents and contains phenolic hydroxyl groups, which can cooperate with Fe to inhibit the production of free radical [31, 32]. This unique structure is the basis of certain physiological functions and pharmacological effects of HD, including suppressing inflammation, regulating diabetes, improving liver fibrosis and protecting cardiovascular systems [32, 33].

#### Effects of HD on hepatic oxidative stress and inflammation

**Fig. 2:**
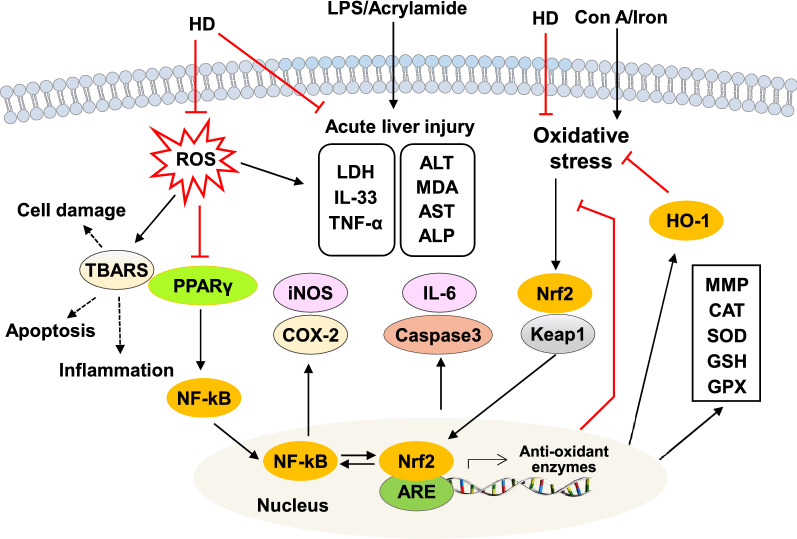
Effects of HD on inflammation and oxidative stress injury. ER stress, endoplasmic reticulum stress; HO-1, heme oxygenase-1; Nrf2, nuclear factor erythroid 2-related factor 2; PPARγ, peroxisomal proliferator receptor gamma; ROS, reactive oxygen species; TBARS, thiobarbituric acid reactive substances

Stimulation with a variety of pathogenic factors, such as viruses, bacteria, drugs, and chemical poisons, induce excessive oxidative stress and inflammation, which leads to hepatic inflammatory infiltration and the release of proinflammatory cytokines, ultimately causing liver injuries [34, 35]. Recently, HD and its derivatives have been demonstrated as possessing hepatoprotective properties by regulating both oxidative stress and inflammation (Fig. 2). A previous study has shown that HD (40 μM) significantly reversed the tert-butyl hydroperoxide (t-BuOOH)-induced depletion of mitochondrial membrane potential (MMP) and lactate dehydrogenase (LDH) release, thereby reducing the reactive oxygen species (ROS) level by facilitating the phosphorylation of extracellular signal-regulated kinase (ERK)/mitogen-activated protein kinase (MAPK), activating the nuclear translocation of nuclear factor erythroid 2-related factor 2 (Nrf2) and subsequently upregulating heme oxygenase 1 (HO-1) expression in hepatocytes [36]. It has also been indicated that HD (10, 15, 80 and 100 mg/kg) has shown anti-oxidant activity and plays a protective role against acrylamide (AA)-, concanavalin A (Con A)-, iron-, zinc oxide nanoparticle-induced oxidative damage and T cell activation in livers of rats by reducing the serum levels of liver function enzymes (alanine transaminase (ALT), aspartate transaminase (AST), alkaline phosphatase (ALP)) and malondialdehyde (MDA), elevating levels of glutathione (GSH), superoxide dismutase (SOD), catalase (CAT) and glutathione peroxidase (GPx) and reducing the expression and release of high-mobility group box 1 (HMGB1) [37–40]. A later study has also confirmed the anti-oxidant and chemoprotective capacities of HD (50 mg/kg) in liver tissues through the reduction serum levels of progesterone, estradiol, ALT, AST, LDH, urea and creatinine in 7,12-Dimethylbenzanthracene (DMBA)-induced rats model [41].

Previous findings suggest that the anti-inflammatory properties of HD are at least partly dependent on its anti-oxidative stress function because the HD-induced downregulation of inflammatory genes is generally associated with the activation of anti-oxidant pathways. HD (5 mg/kg) has been reported to reduce lipopolysaccharide (LPS)-induced both oxidative stress and inflammation in mice, as indicated by the considerable elevation of GSH and CAT and the reduction of pro-inflammatory cytokines IL-33 and tumor necrosis factor-α (TNF-α) [42]. Nuclear factor-kappa B (NF-κB) and Nrf2 are two crucial transcription factors that regulate the cellular responses to inflammation and oxidative stress, respectively. Additional genetic and pharmacological studies have demonstrated that functional crosstalk between the two important pathways exists, thus revealing that NF-κB activity can be exacerbated by Nrf2, thereby leading to increased cytokine production, whereas NF-κB can modulate Nrf2 transcription and activity [43, 44]. Peroxisomal proliferator receptor gamma (PPARγ) has a key role in the protection against oxidative stress and inflammation through the regulation of Nrf2 pathway and NF-κB activation [45]. A previous study reported that HD (25 mg/kg) inhibited CYP-induced inflammation and lipid abnormalities by upregulating hepatic PPARγ expression and downregulating NF-κB expression [46].

Previous studies have suggested that HD directly regulates inflammatory responses. It has also been reported that several HD derivatives have both anti-oxidant and anti-inflammation activities. Cyclooxygenase 2 (COX-2) is rarely expressed in normal tissues but is induced upon inflammatory cytokines stimulation and aggravates inflammatory response [47]. Similar to COX-2, inducible nitric oxide synthase (iNOS) is an inducible gene expressed during inflammation that synergistically activates NF-κB and promotes cellular inflammation [48]. Shi and colleagues have reported that neohesperidin dihydrochalcone (NHDC) (200 mg/kg), a derivative of HD, shows potent anti-inflammatory and anti-apoptotic effects against PQ-induced acute liver damage in mice by downregulating the expressions of both COX-2 and iNOS [30]. Additionally, as a xenobiotic agent, CCl_4_ administration has been shown to increase oxidative stress and lipid peroxidation damage in the liver. Further studies found that NHDC relieved oxidative stress and inflammation in hepatocytes. NHDC treatment not only downregulated the protein expressions of NF-κB, interleukin-6 (IL-6), caspase 3 and caspase 8 in livers of CCl_4_-induced mouse model but also increased cell viability, decreased intracellular levels of ROS and thiobarbituric acid reactive substances (TBARS) in HepG2 cells [28]. Meanwhile, NHDC prevented CCl_4_-induced oxidative damage and lipotoxicity through the activation of Nrf2/ARE-meditated free radical scavenging responses, inhibition of Nrf2 ubiquitination and upregulation of c-Jun N-terminal kinase (JNK) and p38 [49].

#### The effects of HD on hepatic lipid metabolism

**Fig. 3:**
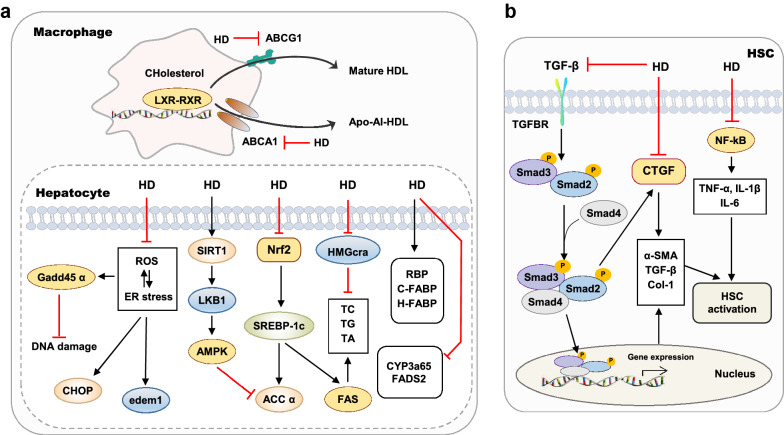
Effects of HD on hepatic lipid metabolism (**a**) and liver fibrosis (**b**). ABCG8, ATP-binding cassette transporters G8; ACC α, acetyl coenzyme A carboxylase alpha; C-FABP, cutaneous fatty acid-binding protein; CTGF, connective tissue growth factor; H-FABP, heart fatty acid-binding protein; LXR, liver X receptor; RBP, retinol-binding protein; RXR, retinoid X receptor; SIRT1, sirtuin 1

Lipid metabolism disorders are abnormalities of lipids and their metabolic substances in the blood and other tissues caused by congenital or acquired factors. The incidence rates of lipid metabolism disorders in the liver, mainly including non-alcoholic fatty liver disease (NAFLD) and non-alcoholic steatohepatitis (NASH), continue to rise worldwide. Recently, HD has been reported to have a therapeutic effect on lipid disorders in the liver (Fig. 3a). Emerging evidence indicated that HD (0.08% in diet) improved hypercholesterolemia and fatty liver injury by inhibiting both the synthesis and absorption of cholesterol, and regulating the mRNA expression of retinol-binding protein (RBP), cutaneous fatty acid-binding protein (C-FABP), and heart fatty acid-binding protein(H-FABP) [50]. It has been reported that HD (0.05% in diet) demonstrates beneficial effects in obese cats by lowering the concentrations of plasma acid glycoprotein and haptoglobin and the mRNA levels of interferon-gamma (IFN-γ) and IL-2 in the liver [51]. High-fat diet (HFD) resulted in increased insulin resistance and lipid accumulation, accompanied by the dysregulation of the oxidative stress response. HD (100 mg/kg) reduced lipid accumulation by decreasing levels of acetyl coenzyme A carboxylase α (ACC α) and fatty acid synthase (FAS), upregulating expressions of hepatic ATP-binding cassette transporters G8 (ABCG8), macrophage ATP-binding cassette transporters A1 (ABCA1) and G1 (ABCG1) and normalizing activities of anti-oxidant enzymes in the HFD-fed LDL receptor-deficient (LDLR^−/−^) mice [52]. Previous studies have demonstrated that the activation of sirtuin 1 (SIRT1) promotes the expression of its downstream liver kinase B1 (LKB1) and AMP-activated protein kinase (AMPK), inhibits the expression of FAS and ACC and further prevents fat deposition in the liver [53]. As reported, HD (100 mg/kg) markedly improved hepatic lipid deposition and oxidative stress by decreasing NF-κB levels, increasing SIRT1 levels and upregulating SOD and CAT activities in streptozotocin (STZ)-induced rat model [54].

Alcohol is mainly metabolized in the liver and is an important cause of lipid metabolism disorders. In addition to oxidative stress, alcohol-induced activation of the endoplasmic reticulum (ER) stress pathway also plays a pivotal role in the pathogenesis of alcoholic liver disease (ALD). ER stress promotes the hydrolysis of the fat synthesis transcription factor, sterol regulatory element-binding protein 1C (SREBP-1c), thereby leading to the induction of fat synthase expression and ultimately resulting in lipid metabolism disorder [55]. HD (12.5 μg/ml) has also been documented as preventing ER stress and DNA damage, inhibiting zebrafish larvae-induced alcoholic lipid accumulation by suppressing the expressions of lipid metabolism-related genes, including cytochrome P450 family 3 subfamily A polypeptide 65 (CYP3a65), HMG coenzyme A reductase a (HMGcra), FAS and fatty acid desaturase 2 (FADS2), and decreasing the levels of the ER stress-related genes, including DNA damage-inducible transcript 3 (CHOP), growth arrest and DNA damage-inducible α a (gadd45 α) and ER degradation-enhancing α-mannosidase-like protein 1 (edem1) [56]. Additionally, glucosyl hesperidin (G-HD), another HD derivative, and caffeine are effective for controlling hepatic lipogenesis and obesity by inhibiting the hepatic expression of SREBP-1c and FAS [57].

#### The role of HD in liver fibrosis

Bile acid accumulation, hepatocyte damage and following inflammation and immune response promote the activation of hepatic stellate cell (HSC), accumulation of extracellular matrix (ECM), collagen deposition and eventually leads to liver fibrosis [58]. Recently, there are growing interests in the protective effects of HD and other ingredients derived from rutaceae plants on the progression of liver fibrosis (Fig. 3b). A previous study has demonstrated that HD (100 mg/kg) ameliorates liver fibrosis and decreases the levels of ALT, AST, ALP as well as hepatic MDA content and iNOS gene expression, α-smooth muscle actin (α-SMA) and caspase-3 in dimethylnitrosamine (DMN)-induced rat model [59]. Transforming growth factor-β1 (TGF-β), one of the master mediators of liver fibrosis, binds to TGF receptors on the surface of HSC and activates the small mothers against decapentaplegic (Smad) homolog pathway, which in turn activates HSC, promotes the synthesis and secretion of ECM and results in liver fibrosis [60]. HD (50 mg/kg) relieved liver fibrosis by reducing TBARS formation and caspase-3 activation and inhibiting the expression of NF-κB, TGF-β and connective tissue growth factor (CTGF) in the CCl_4_-induced fibrotic model [61, 62]. A recent study has further demonstrated that HD inhibits HSC proliferation and activation by reducing the expression of TGF-β and TGF-β-mediated Smad2, Smad3 and CTGF pathways [63].

In addition to its use as a single-agent, another study has also shown that HD in combination with taurine alleviates CCl_4_-induced cholesterol accumulation, cholestasis and liver fibrosis [64]. When used concurrently with diethylcarbamazine (DEC), HD (200 mg/kg) attenuated alcohol-induced fibrogenesis and blunted necroinflammation in HSCs by reducing α-SMA expression and inhibiting TGF-β1 and NF-κB pathways [5]. Morsy and colleagues designed an HD-loaded surface-modified liposome formulation and further conjugated it with a homing ligand recognized by HSCs. As expected, this advanced carrier system predominantly increased the uptake of HD in the liver and improved liver fibrosis in a rat model [65].

#### Effects of HD on liver cancer

As the most common cause of death worldwide, liver cancer is still a major clinical challenge due to poor diagnosis and various risk factors, including virus, alcohol, smoking, toxicant and other factors. The anti-cancer effects of HD and its derivatives have been extensively studied. It has been reported that HD (50 μM) suppresses human hepatic cancer (HepG2) cell proliferation by inhibiting matrix metalloproteinase-9 (MMP-9) enzymatic activity, inhibiting phosphorylation of p38 and JNK signaling pathways and suppressing the NF-kB and activator protein 1 (AP-1) pathway [66]. The B-cell lymphoma-2 (Bcl-2) family of apoptotic cell death regulators includes both anti-apoptotic proteins (such as Bcl-2 and Bcl-xL) and pro-apoptotic proteins (such as Bax and Bak) [67]. Recently, researchers found that HD treatment (50 μM) inhibited the proliferation and induced the apoptosis of HepG2 cells, which was accompanied by the downregulation of Bcl-xL protein and the upregulation of Bcl-2-associated X (Bax), Bcl-2 antagonist killer (Bak) and truncated Bid (tBid) [68]. Naz et al. further reported that HD (5 mg/ml) inhibited proliferation and induced apoptosis of HepG2 cells by upregulating the Bax protein level and activating caspase-3-dependent intrinsic pathway [69]. The wingless/integrated (Wnt) pathway is a key regulator of cell survival, proliferation, migration and invasion, especially in cancer progression [70]. HD (150 mg/kg) significantly prevented thioacetamide (TAA)-induced inflammation, oxidative imbalance and hepatocarcinogenesis by downregulating the expression of Wnt3a, β-catenin, Cyclin D1 and Wnt5a in rats [71]. Interestingly, another study has proposed that HD is a candidate epigenetic regulator in the regulation of tumoral biology. HD (0.78 mM) induced hypomethylation on the LINE-1 sequence (up to 47% hypomethylation at 12.5 mM) and the ALU-M2 repetitive sequences (up to 32% at 6 mM) in HL60 tumor cells and thus inhibited liver tumor growth in the diethyl nitrosamine-induced rat model [72].

In addition to directly targeting cancer cells in vitro, recent studies have also suggested that HD inhibits hepatocarcinogenesis in various animal models. It has been documented that HD (50 mg/kg) suppresses cell proliferation, collagen deposition and further inhibits hepatocarcinogenesis by suppressing NF-κB, TGF-β1/Smad3 signaling and activating Nrf2/ARE/HO-1 and PPARγ pathways in diethylnitrosamine (DEN)/CCl_4_-induced rats model [73]. Evidence also suggested that HD (200 mg/kg) inhibited the DEN-induced liver fibrosis and tumor growth through the upregulation of phosphatidylinositide 3-kinases (PI3K), serine/threonine kinase (AKT), cyclin-dependent kinase 2 (CDK-2) signaling pathway [74]. Most recently, emerging evidence has emphasized the role of long noncoding RNA (lncRNA) and exosomes in the progression of liver fibrosis and HCC. Hasanin and colleagues first reported that HD (100 mg/kg) significantly suppressed liver carcinogenesis in the DEN- and 2-acetylaminofluorene-induced rat cancer model via decreasing exosomal RAB11A and lncRNA-RP11-583F2.2 and increasing exosomal miR-1298 in rat liver tissues [75].

### Naringenin

**Fig. 4:**
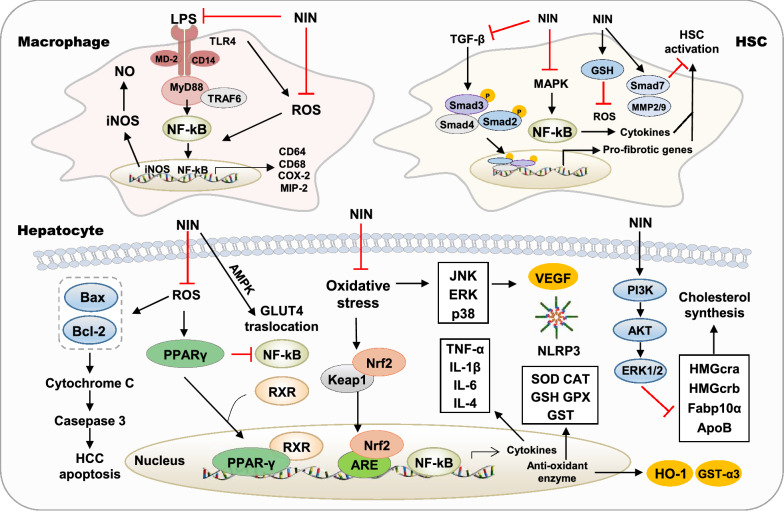
Molecular targets of NIN in liver diseases. ApoB, apolipoprotein B; GLUT4, glucose transporter type 4; MIP-2, macrophage inflammatory protein 2; TLR4, toll-like receptor 4

NIN, also called 4′,5,7-trihydroxy flavanone, is predominantly found in *Citrus* fruits and has various pharmacological activities [76]. After oral administration, most NIN undergoes bacteriostatic metabolism, while the rest is reabsorbed into the blood. The effect of NIN on various liver diseases and possible molecular mechanisms of action are summarized below (Fig. 4).

#### The effect of NIN on oxidative stress

Recently, the hepatoprotective effects of NIN in the chemicals- and toxicant-induced liver injury have been investigated. Acetaminophen (APAP) overdose is the most frequent cause of drug-induced acute liver failure. N-acetyl-p-benzoquinone imine (NAPQI), an active metabolite of APAP, induces the depletion of intracellular and mitochondrial GSH in the liver, triggers excessive oxidative stress and results in massive hepatocytes necrosis [77]. Emerging evidence suggested that NIN (400 mg/kg) protected APAP intoxication in various animal models as indicated by decreased levels of serum ALT, AST, LDH and liver MDA. A significantly increased ratio of GSH/oxidized glutathione ratio (GSSG), indicating the activation of the defense mechanisms against oxidative stress, was observed in this study [78]. Additionally, the anti-oxidative effects of NIN have been demonstrated in numerous chemical-induced liver injury animal models. As reported, NIN (50 mg/kg) significantly attenuated oxidative stress by decreasing the levels of LDH, TBARS and LOOH, upregulating the activities of alcohol dehydrogenase (ADH), aldehyde dehydrogenase (ALDH), SOD and CAT and recovering ethanol-induced depletion of GSH in the mouse model [79]. NIN (50 mg/kg) also significantly prevented CCl_4_-induced oxidative stress, steatosis and altered ultrastructure of hepatocytes by upregulating anti-oxidant enzyme activities (SOD, GPx and GSH) in mice model [80]. After methotrexate (MTX) administration, NIN (50 mg/kg) significantly increased the levels of GSH, as well as CAT, SOD, and GPx expression, and thus decreased the hepatic levels of MDA, NO, TNF-α and IL-6 in rats [81]. Previous studies also demonstrated that NIN (50 mg/kg) restored the levels of enzymatic and non-enzymatic anti-oxidants, including SOD, CAT, GPx, GST, GSH, vitamin C and E and prevented cadmium-caused lipid peroxidation by lowing the levels of TBARS and lipid hydroperoxides [82]. Similarly, the pretreatment of rats with NIN (50  mg/kg) markedly potentiated anti-oxidant efficacies and TBARS and further reduced arsenic-induced oxidative stress and decreased lipid peroxidation in arsenic-induced rats [83].

It is noteworthy that almost all studies have consistently found NIN-induced elevation of GSH levels and the upregulation of anti-oxidant proteins, including GPx, SOD and CAT. However, the underlying mechanisms remain elusive. Until recently, a study has found that NIN (50 mg/kg) alleviates liver injury by increasing the hepatic expression of the transcription factor Nrf2, which in turn activates the expression of the anti-oxidative enzyme genes HO-1 and glutathione S-transferase alpha 3 (GST-α3) in a CCl_4_-induced hepatotoxic rat model [84].

#### The role of NIN in the hepatic inflammation

It has been reported that NIN (50 mg/kg) significantly prevents high cholesterol diet-induced macrophage infiltration and inflammation in the rat model, as indicated by decreased plasma fatty acid and hepatic levels of pro-MMP-2/9, NF-κB and pro-inflammatory cytokines, including TNF-α, IL-6 and IL-1β [85]. Most recently, NIN (50 mg/kg) has also been found to improve hepatic inflammation and lipid peroxidation by decreasing the hepatic levels of TNF-α, IL-6, NF-κB, COX-2, macrophage inflammatory protein 2 (MIP-2), iNOS and CD14 in ethanol-fed rats [86]. NIN (30 μM) also significantly inhibited inflammatory responses by blocking LPS-induced NO production, iNOS levels and NF-κB activation in murine J774 macrophages [87]. In addition, researchers have administered NIN (1% and 3% in diet) to mice with gestational diabetes mellitus symptoms and found that NIN and NRG significantly decreased TNF-α-induced ROS production, enhanced glucose transporter type 4 (GLUT4) membrane translocation, inhibited inflammation in the liver and improved diabetes. These effects were abrogated when AMPK was inhibited, thereby suggesting a potential role of AMPK activation in NIN (50 μg/ml)-induced anti-inflammation effects [88]. Further studies are required to elucidate the mechanisms underlying the anti-inflammation activities of NIN in addition to its anti-oxidative stress properties.

#### The effect of NIN on fatty liver diseases

The protective effects of NIN against fatty liver diseases are not fully characterized. The overexpression of apolipoprotein B (ApoB) is associated with lipid accumulation and fatty liver diseases. A previous study has documented that NIN (25 μM) treatment stimulates intracellular ApoB degradation and inhibits ApoB secretion by activating PI3K and ERK1/2 in hepatocytes [89]. Another study suggested that NIN (5 mg/l) attenuated lipid accumulation, inhibited ER stress and prevented alcoholic-induced morphological changes associated with ER stress by lowering apoptosis and DNA damage and decreasing the expression of alcohol and lipid metabolism-related genes, including CYP family 2 subfamily Y polypeptide 3 (CYP2y3), CYP3a65, HMGcra, HMGcrb, FAS, FABP10α, FADS2 and enoyl-CoA hydratase, short chain 1 (echs1) in zebrafish model [90]. Furthermore, it has been reported that NIN administration (100 mg/kg) decreases hepatic lipid accumulation and reduces the expression of CD68 and CD64 by inhibiting the nucleotide-binding oligomerization domain-like receptor family pyrin domain-containing 3 (NLRP3)/NF-κB signaling pathway in livers of MCD-fed wild-type (WT) mouse model, but these changes have been found to be less effective in livers from NLRP3^−/−^ mice. Besides, NIN suppressed the elevated expression of NLRP3 caused by LPS and inhibited the activation of Kupffer cells [91].

#### The effect of NIN on liver fibrosis

A previous study has demonstrated that NIN prevents HSC transdifferentiation, collagen synthesis and deposition and ameliorates hepatic fibrosis in rats by downregulating the TGF-β, MAPK and Toll-like receptor (TLR) pathways [92]. In addition, NIN (100 mg/kg) decreased collagen deposition and showed anti-fibrosis effects on CCl_4_-induced rat model by decreasing the expression of TGF-β, α-SMA, CTGF, collagen type 1 (Col-1) and MMP-13, preventing NF-κB activation and the subsequent production of IL-1 and IL-10. These effects might be mediated by the NIN-mediated inhibition of JNK activation, which prevents Smad3 phosphorylation in the linker region by JNK [93]. Recent studies have also shown that NIN (100 mg/kg) directly reduces JNK activation and Smad3 phosphorylation, restores normal GSH, MMP-9 and MMP-2 activities, lowers α-SMA expression and thus improves liver fibrosis in the CCl_4_-induced rat model [94].

#### The effect of NIN on hepatic carcinoma

A growing number of studies have suggested that NIN is a potent anti-cancer agent. It has been reported that NIN (50 μM) inhibits cell proliferation and promotes apoptosis by triggering the mitochondrial-mediated apoptosis pathway, increasing the ratio of Bax/Bcl-2, activating caspase-3, and inducing cytochrome C release in HepG2 cells [95]. Ahmed et al. employed the DEN/2-acetylaminofluorene (2AAF) rat HCC model to investigate the anti-cancer activity of NIN in vivo and found that NIN treatment significantly abated hepatocarcinogenesis by decreasing hepatic NO levels and lipid peroxidation, upregulating SOD, GPx and CAT, and increasing intrahepatic GSH content. NIN (10 mg/kg) treatment also significantly promoted expressions of anti-inflammatory cytokines IL-4, and tumor suppressor P53 in livers of these rats [96]. Most recently, it has been reported that 6-C-(E-phenylethenyl) NIN (6-CEPN), a novel seminatural derivative of NIN, inhibits HCC tumor growth and enhances the sensitivity of HCC cells to therapeutic drugs, including cisplatin, 5-fluorouracil, and sorafenib. The upregulation of glycogen synthesis kinase (GSK) 3β and the downregulation of Wnt/β-catenin signaling, by inducing β-catenin degradation and inhibiting its nuclear translocation, was crucial for 6-CEPN-induced anti-cancer effects [97, 98].

### Nobiletin

**Fig. 5:**
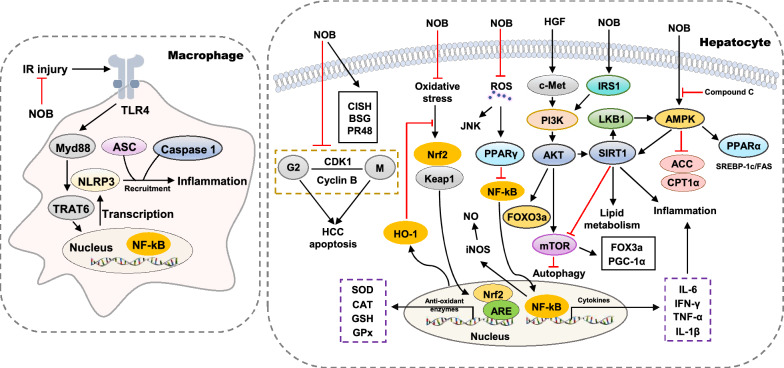
Molecular targets of NOB in liver diseases. BSG, basigin; CISH, cytokine-inducible SH2-containing protein; CPT1α, carnitine palmitoyltransferase 1α; FOXO3 a, forkhead box O3 a; PGC-1α, peroxisome proliferator-activated receptorγcoactivator-1α

NOB, also known as tangerine flavin, is a polymethyl flavonoid ingredient that has neuroprotective, anti-inflammatory, anti-oxidant and hepatoprotective effects (Fig. 5).

#### The effect of NOB on oxidative stress and liver inflammation

Various studies have suggested that NOB may be responsible for the anti-inflammatory effects of *Citrus* peels and have therapeutic effects on various liver injury models. NOB (75 μM) markedly suppressed iNOS expression and NO production, reduced the transcriptional activity of iNOS promoter-luciferase constructs and the DNA-binding activity of nuclear NF-κB in the IL-1β-treated hepatocytes [99]. Recently, it has been reported that NOB (100 mg/kg) attenuates LPS/D-galactosamine (GalN)-induced liver inflammation by upregulating the Nrf2 anti-oxidant pathway and subsequently downregulating the production of NF-κB-mediated pro-inflammatory cytokines [100]. Similar to other ingredients from AFI and AF, NOB (10 mg/kg) also reversed oxidative stress and inflammation caused by APAP by lowering the levels of MDA, IL-1β, IL-6, and TNF-α, increasing GSH-Px activities and Nrf2 and HO-1 signaling pathways in a rat model [101]. Most recently, pretreatment with NOB (10 mg/kg) was reported to attenuate Con A-induced liver inflammation by lowering serum liver enzymes, decreasing ROS, and suppressing the release of inflammatory cytokines such as TNF-α and IFN-γ. In addition, NOB (1 μM) promoted proliferation and alleviated apoptosis of hepatocytes by suppressing JNK activation [102].

Furthermore, Wu et al. explored the protective effects of NOB against IR injury after liver transplantation and found that NOB (50 mg/kg) inhibited the activation of TLR4/NF-κB signaling pathway and the downstream expression of inflammatory mediators in activated Kupffer cells, which then decreased the hepatic infiltration of macrophages and CD4^+^ lymphocytes, and suppressed liver inflammation [27]. Recently, it has also been reported that NOB (5 mg/kg) downregulates autophagy-regulatory gene expression, increases forkhead box O3 a (FOXO3a) expression and its nuclear translocation by activating SIRT-1, AKT and PPARγ coactivator-1α (PGC-1α) pathways, which in turn attenuates hepatocyte apoptosis and inflammation in mice with IR injury [29]. These findings suggest that NOB may possess therapeutic potential for treating liver IR injury or have protective property for liver preservation during transplantation.

#### The effect of NOB on lipid metabolism

It has been documented that NOB (25 μM) reduces the expression of SREBP-1c and FAS and attenuates high glucose-induced lipid accumulation in HepG2 hepatocytes by increasing the phosphorylation of AMPK and ACC, which is abrogated by compound C, an AMPK inhibitor [103]. Interestingly, another study has suggested that NOB (200 μM) enhances glucose uptake by stimulating insulin receptor substrate 1 (IRS1)/Akt signaling pathway, and attenuates palmitate-induced lipogenesis in HepG2 cells via the brain and muscle arnt-like protein-1 (Bmal1)-dependent modulation of AMPK-Sirt1 signaling pathway and the suppression of de novo lipogenesis genes [104]. A recent in vivo study demonstrated that low-dose NOB (0.02% in diet) attenuated HFD-induced lipid droplet accumulation, hepatic inflammation and NAFLD by increasing the expression of hepatic fatty acid oxidation-related genes, including PPARα and carnitine palmitoyltransferase 1 α (CPT1α) [105]. The liver and gut exchange nutrition, bile acids, metabolites and microbial products multiple times a day. Emerging studies highlight the importance of hepato-enteral circulation in understanding chronic liver disorders, especially metabolic liver diseases [106]. A previous study indicated that NOB (25 μM) prevented intestinal injury caused by excessive fructose intake by suppressing the uptake of fructose but not of l-leucine or glycylsarcosine [107], thus suggesting that NOB may protect the liver from fructose-induced lipid accumulation by reducing the intestinal absorption of fructose. This finding suggests a potential regulatory role of NOB in hepatic lipid metabolism by targeting intestine, although further experimental evidence is required.

#### The effect of NOB on hepatocarcinogenesis

Previous studies have suggested that NOB is a potential therapeutic agent targeting against HCC. Microarray analysis from Ohnishi et al. indicated that NOB specifically upregulated the expression of a set of genes involved in the progression of HCC growth, including cytokine-inducible SH2-containing protein (CISH), basigin (BSG) and protein phosphatase 2A 48 kDa regulatory subunit (PR48). The administration of NOB (1 mM) significantly inhibited the proliferation of HCC cells and induced G2/M cell cycle arrest and apoptosis [108]. Furthermore, Shi et al. found that NOB (2.5 μM) also significantly reduced hepatocyte growth factor (HGF)-induced cell invasion and migration of HepG2 cells by suppressing phosphorylation and membrane localization of c-Met, Akt, and ERK 2 [109].

### Naringin

NRG (naringenin-7-*O*-rhamnoglucoside) is another flavanone glycoside that can be isolated from *AFI* and *AF* as well as from other *Citrus* grandis. Although a large number of studies have routinely investigated the pharmacological effects of NIN and NRG, simultaneously and suggested that they have similar bioactivities, recent advances have revealed unique molecular mechanisms underlying the hepatoprotective effects of NRG.

The anti-oxidative effects of NRG have been found in numerous chemical-induced liver injury animal models. It has been demonstrated that NRG (40 mg/kg)-induced hepatoprotective effect in the APAP-induced model is involved in the upregulation of farnesoid X receptor (FXR) and downregulation of kidney injury molecule (KIM-1) expression [110]. In addition, NRG (20 mg/kg) significantly amended APAP-induced hepatocyte steatosis and inflammation through the reduction of TNF-α levels and liver lipid peroxidation, and enhancing levels of IL-4 and GSH and activities of SOD, GST and GPx in rat livers [111]. Besides APAP, NRG (60 mg/kg) scavenged free radical and exhibited anti-oxidant, anti-inflammation and anti-apoptosis effects by suppressing CYP2E1 expression and MAPK signaling pathway in the CCl_4_-induced mice model [112]. NRG (50 nM) also significantly attenuated oxidative stress by upregulating the activities of GPx, GST, SOD and CAT and recovering iron-induced depletion of GSH in mouse liver mitochondria in vitro [113]. NRG (50 mg/kg) significantly prevented CP-induced inflammation, apoptosis, autophagy, and oxidative DNA damage by upregulating enzyme activities of SOD and GPx, decreasing the levels of TNF-α, NF-κB, IL-6, and IL-1β, and activities of iNOS and COX-2 in rats model [114]. Previous studies also demonstrated that NRG (5 μM) maintained cellular redox homeostasis and suppressed CdCl_2_-mediated lipotoxicity by upregulating the endogenous activities of SOD and CAT and reducing caspase 3 cleavage and cytochrome c release [115]. Additionally, NRG (40 mg/kg) pretreatment markedly increased the GPx and SOD activities in mice, which further alleviated mitomycin C (MMC)-induced oxidative stress and decreased lipid peroxidation [116]. A recent study found that NRG (100 mg/kg) alleviated liver injury by increasing hepatic expression of transcription factor Nrf2 and its activated Nrf2-mediated expression of antioxidative enzyme genes HO-1, SOD and CAT, suppressing NF-κB activity and decreasing inflammatory cytokines TNF-α and IL-6 in a perfluorooctane sulfonate (PFOS)-induced hepatotoxic mouse model [117].

Previous studies have suggested that NOB is a potential therapeutic agent against hepatic inflammation. It has been reported that NRG (100 mg/kg) prevents 5-fluorouracil (5-FU)-induced hepatotoxicity and inflammation in the rat model, as indicated by significantly decreased serum levels of AST, ALT, ALP and LDH as well as decreased hepatic levels of IL-6, TNF-α and IL-1α [118]. Furthermore, it has been reported that NRG (40 mg/kg) administration protects STZ-induced diabetes, oxidative stress and liver damage by decreasing the NF-κB/IL-6/COX-2 expression and blocking iNOS/NO/nitrosylated protein pathway [119]. In vitro study further reported the anti-inflammation effects of NRG in RAW 264.7 macrophages and reported that NRG (6.4 μM) directly inhibited macrophage activation and inflammatory responses by blocking LPS-induced NO production and oxidative stress [120].

Hepatic inflammation plays a critical role in the progression of almost all liver diseases, from fatty liver to severe liver fibrosis. NRG treatment (200 mg/kg) inhibited fatty acid synthesis, increased fatty acid oxidation and further attenuated HFD-induced fatty liver injury and insulin resistance, as indicated by the downregulated expression of FAS, ACCα and PPARγ in the mouse model. These NRG-induced inhibitory effects on hepatic inflammation and insulin resistance was mediated by phosphorylating AMPKα and IRS1 [121]. A previous study demonstrated that NRG (40 mg/kg) ameliorated arsenic-induced hepatic damage and fibrosis in rats through the alleviation of elevated oxide-nitrosative stress and downregulation of caspase-3, TGF-β and TNF-α levels [122]. Recently, Shi and colleagues applied HPLC analysis and found that NRG (20 ng/mL) concentration prevented HSC activation through the inhibition of mammalian target of rapamycin (mTOR) pathway and induction of autophagy [123]. Consistently, another study suggested that NRG (40 mg/kg) not only inhibited PI3K/AKT and following mTOR pathway but also increased the number of caspase-3 positive cells in fibrotic areas, decreased pro-inflammatory factors and alleviated experimental liver fibrosis induced by TAA [124]. Similar to other ingredients isolated from AFI and AF, NRG (20 μM) triggered human HepG2 cell apoptosis via activating caspase 9 and caspase 8, increasing the expression of Bax and Bak and decreasing expression of Bcl-extra large (Bcl-xL) [125].

### Tangeretin

TN, also named as 5,6,7,8-Tetramethoxy-2-(4-methoxyphenyl)-4-benzopyrone, is one of the compounds, responsible for the medicinal effects of AFI and AF, including anti-oxidative stress, anti-inflammation and anti-tumor effects [13]. It has been reported that TN exerts strong inhibitory effects on the expression of fatty acid generating and transporting proteins, such as glucose-6-phosphate dehydrogenase (G6PD), FABP4, LPL and CD36/FAT, CYP27A1, as well as transcription factors, including FXR, PPARγ, and LXR, and in turn, significantly attenuates hepatic inflammation and lipid accumulation in mice with NASH [126]. Pretreatment with TN (50 mg/kg) markedly attenuated the cisplatin-induced lipid metabolism dysregulation and liver injury by suppressing the expression of TNF-α and Bax, and activation of p38, JNK and ERK1/2 in the liver [127]. A further study reported that TN (5 μM) protected against t-BuOOH-induced oxidative stress and liver injury, as indicated by increased GSH, decreased levels of ROS and LDH and the activation of anti-oxidant pathways, HO-1 and NAD(P)H quinone oxidoreductase 1 (NQO1), which was dependent on Nrf2/ARE pathway activation [128].

Autophagy is an intracellular degradation and recycling process that exerts a striking effect on hepatic lipid metabolism and fibrosis. Autophagy-related (Atg) protein, LC3-II including LC3a and LC3b, Beclin-1, autophagy substrate p62 and cathepsin B (CTSB) play significant roles in autophagosome formation [129]. In addition to relieving lipid accumulation and inflammation during liver injury, TN (30 ug/ml) has been found to activate autophagy, as indicated by decreased p62 expression and increased LC3II/LC3I ratio, and to further prevent the migration and proliferation of HepG2 cells. Additionally, TN activated JNK1/Bcl-2 pathway and disturbed the interaction between Bcl-2 and BECLIN1 [130]. Interestingly, the knockdown of BECLIN1 partially alleviated the TN-induced hepatoprotective effects in HepG2 cells. These findings help delineate the mechanisms underlying the TN-induced inhibition of HCC development.

### Hesperetin

HT (3′,5,7-trihydroxy-4′-methoxyflavanone) is a dihydro flavonoid extracted from the fruits of *Citrus* plants. Numerous studies have reported promising therapeutic effects of HT against liver injury by inhibiting inflammation, oxidative stress, and cancer metastasis both in vitro and in vivo. It has been documented that HT (100 mg/kg) ameliorates liver damage through the inhibition of NF-κB expression and following TNF-α production in blunt chest trauma rat model [131]. As a commonly used liver fibrosis model, bile duct ligation (BDL) induces bile duct obstruction and leads to experimental cholestasis and periportal fibrosis. Another study demonstrated that HT treatment effectively prevented the progression of hepatic inflammation and liver fibrosis in the BDL mouse model. HT (200 mg/kg) not only inhibited the expression of pro-inflammatory cytokines TNF-α and IL-6, but also suppressed HSC activation and ECM formation through the inhibition of TGF-β1/Smad pathways [132]. Most recently, HT (5 mg/kg) was reported to mitigate oxidative stress, inhibit hepatic macrophages and neutrophils recruitment by blocking p38, p65 and TLR-4 activation in APAP-induced mice model. Furthermore, in vitro experiment revealed that HT (3 μM) markedly alleviated hepatocyte apoptosis and inflammation through the upregulation of HO-1 expression. The knockdown of HO-1 by HO-1 siRNA reversed these hepatoprotective effects of HT on APAP-induced liver injury in vivo and in vitro [133]. Additionally, several derivatives of HT have anti-inflammation and anti-fibrosis activities. HT derivative-14 activated PPARγ and subsequently inhibited the expression of phosphor-Janus kinase-1 (JAK1) and phosphor-signal transducer and activator of transcription 1 (STAT1) in LPS-treated RAW264.7 cells, thus blocking JAK1-STAT1-mediated inflammatory responses [134]. Li and colleagues further demonstrated that HT derivative 11 [6-(4-morpholine-2-ethylpiperazinyl) hesperidin] inhibited the activation and proliferation of HSCs by remarkably inhibiting PI3K/AKT pathway and upregulating phosphatase and tension homologue deleted on chromosome ten (PTEN) in vivo and in vitro [135]. In addition, 4-Methylcoumarin-[5,6-g]-HT, an HT derivative produced by structural modification, significantly suppressed the release of inflammatory cytokines such as IL-6 and TNF-α and alleviated liver inflammation by inhibiting the phosphorylation of NF-κB p65 and promoting PPARγ activation in the ethanol-fed mouse model [136].

### Eriodictyol

ED, also called (S)-2-(3,4-dihydroxyphenyl)-2,3-dihydro-5,7-dihydroxy-4-benzopyrone), is a dietary flavonoid isolated from rutaceae plants [137]. It has been reported that ED (40 mg/kg) exhibits a protective effect on As_2_O_3_-induced oxidative stress and liver injury by activating Nrf2 and HO-1 signaling pathway-dependent anti-oxidant responses [138]. ED (200 mg/kg) also attenuated APAP-induced hepatotoxicity in heterozygous UDP-glucuronosyltransferase (Ugt1^+/−^) mice but not in their WT littermates by inhibiting hepatic CYP2E1 and CYP3A11 activities and restoring intrahepatic GSH levels by enhancing GPx, GR, GST and SOD activities [139]. A present study found that ED supplementation (0.005% in diet) markedly alleviated HFD-induced adiposity and dyslipidemia by inhibiting lipogenesis and promoting fecal lipid excretion. The downregulation of SREBP1 and FAS and the inhibition of malic enzyme (ME), phosphatidate phosphohydrolase (PAP) and FAS also contributed to improved hepatic steatosis induced by ED. Moreover, ED (0.005% in diet) significantly reduced the plasma levels of pro-inflammatory cytokines, including plasminogen activator inhibitor-1 (PAI-1), IFN-γ and IL-1β, as well as adipokine leptin in HFD-induced mice [140].

### Other flavonoids derived from AFI and AF

In addition to the ingredients discussed above, some flavonoids isolated from AFI and AF also play hitherto unrecognized roles in the treatment of various liver diseases, such as poncirin and narirutin. Findings suggest that poncirin treatment attenuates extrinsic pathway-mediated apoptosis, possibly making it a hepatoprotective agent against toxins. It has also been reported that poncirin (30 mg/kg) significantly attenuates hepatic inflammation and liver injury in complete freund’s adjuvant (CFA)-induced pain mouse model by inhibiting the production of pro-inflammatory cytokines, such as TNF-α, IL-1β, and IL-6, and enhancing the activation of anti-oxidant pathways including Nrf2 and HO-1 [141]. Park et al. reported that narirutin alleviated alcohol-induced liver damage in mice through the prevention of lipid droplet formation, inhibition of NF-κB pathways, and suppression of pro-inflammatory cytokines, TNF-α and IL-1β [142].

## Conclusion and future opportunities

Compared to traditional single-target drugs, bioactive natural ingredients derived from herbs may provide additional benefits in the prevention of chronic diseases with improved efficacy and lower toxicity, and they represent an important source of drug discovery. AFI and AF are commonly used herbal products in TCM and have been prescribed in many TCM formulas for the treatment of various diseases. AFI and AF are both the fruits of rutaceae plant and are harvested at different stages of fruit growth. Interestingly, they are used as distinct medicinal materials in the clinical application of TCM due to their divergent pharmacological effects. However, several obstacles have limited the clinical application of flavonoids extracted from AFI and AF and delayed the discovery of new therapies based on AFI, AF and their derivatives. A recent study has suggested that harvesting times may affect the content and composition of bioactive ingredients in AFI and AF, which causes difficulty in quality control and clinical efficacy evaluation [12]. Specifically, further studies have demonstrated that the amount of total alkaloids, total flavonoids, synephrine, HD and NHDC show a decreasing trend, while the amount of NRG and narirutin increase initially and then decrease during the course of the harvesting season from May to July [143]. These findings indicate that the current quality control standards for AFI and AF, which are designed based on only a few compounds, make it difficult to fully reflect the cumulative changes of secondary metabolites in AFI and AF. Thus, it is necessary to examine the dynamic changes of individual bioactive ingredients isolated from AFI and AF harvested at different phenological stages to establish a reference for quality evaluation.

Although previous clinical studies have reported the pharmacological effects and mechanisms of action of the ingredients isolated from AFI and AF, there is no review focusing on the significance of individual bioactive ingredients in the treatment of liver diseases. Flavonoids extracted from AFI and AF inhibited liver inflammation, reduced oxidative stress damage, suppressed lipid accumulation and further protected the liver from fibrosis and lipotoxicity. The activation of the Nrf2/HO-1 pathway, the downstream upregulation of anti-oxidant enzymes, including SOD, GST and GPx, and the increased intracellular levels of GSH have been proposed as common mechanisms underlying the anti-oxidant effects of these compounds. Moreover, most of the studies have suggested that the reversal of oxidative stress plays a critical role in the anti-inflammation effects of these flavonoids. Several studies have further revealed that the master anti-oxidant regulator Nrf2 is the direct target of flavonoids from AFI and AF. However, it is still unclear how these ingredients trigger Nrf2 signaling. The direct interaction between Nrf2 with these flavonoids needs to be further explored using molecular and biochemical approaches such as luciferase reporter assays, electrophoretic mobility shift assays and chromosome immunoprecipitation analyses.

Although almost all these flavonoids improve hepatic oxidative stress, liver inflammation and lipid metabolism regardless of their etiologies, the minimum effective dose and efficacies vary considerably among diseases. The varying therapeutic efficacies of these flavonoids is largely due to their unique chemical structure or their binding to different target receptors. Generally, as the most studied ingredient, HD (10 mg/kg) is more potent than other flavonoids against toxins- and drugs-induced oxidative damage and lipid peroxide in vivo. Additionally, as little as 50 mg/kg of HD is sufficient to alleviate liver fibrosis from several causes, including the inhibition of NF-κB/IL-1β/IL-10 and TGF-β/Smad2/3/CTGF signaling pathway and prevention of HSC activation. On the other hand, NIN (10 mg/kg) is the most promising anti-cancer candidate compound, as it inhibits cell proliferation and promoted apoptosis by triggering the mitochondrial-mediated apoptosis pathway and decreasing lipid peroxidation and inflammation. These effects can be explained in part by the NIN-induced lipid-lowering activity and the upregulation of hepatic PPARα. Furthermore, both HT (5 mg/kg) and TN (50 mg/kg) have been reported as the most effective anti-inflammation and anti-lipemic agents. Although key evidence is still missing, it is highly possible that HT alleviates inflammatory responses by attenuating cytokine production and inflammatory cells infiltration, which might be associated with TLR4 inhibition.

HD is the most studied bioactive ingredient but its slow metabolism and low bioavailability is a cause for concern. It has been reported that HD is metabolized to its aglycones (HT) in circulation. Although HD and HT share the same main skeletal structure, their biological activities and metabolic process are distinct. There are almost no general phase I metabolites of HT, but 33 types of phase I metabolites of HD can be identified in the serum after administration. As an orally administered medicine, HD is degraded by gastric acid and various enzymes in the digestive tract with relative ease. Previous studies have also suggested that HD is poorly absorbed in its original form through the phospholipid layer of cell membrane due to the presence of glycosyl group in the C7 position. These shortcomings in metabolism and bioavailability affect the absorption, blood concentration and pharmacological effects of HD in vivo. On the other hand, although the oral administration of HT results in favorable bioavailability, its rapid elimination rate necessitates frequent administration to maintain a stable blood concentration. Recently, in order to improve the bioavailability of HD, cellulose acetate phthalate has been used as an enteric polymer to coat and produce the gastro-resistant microcapsule of HD, which increases the dissolution rate of HD in the intestinal environment and improves its bioavailability [144]. Furthermore, although not reviewed above, recent studies have reported that the alkylation, acylation and formation of complexes with metal ions could be used for producing HD derivatives and improving its pharmacokinetic properties and therapeutic effects to some extent.

**Fig. 6:**
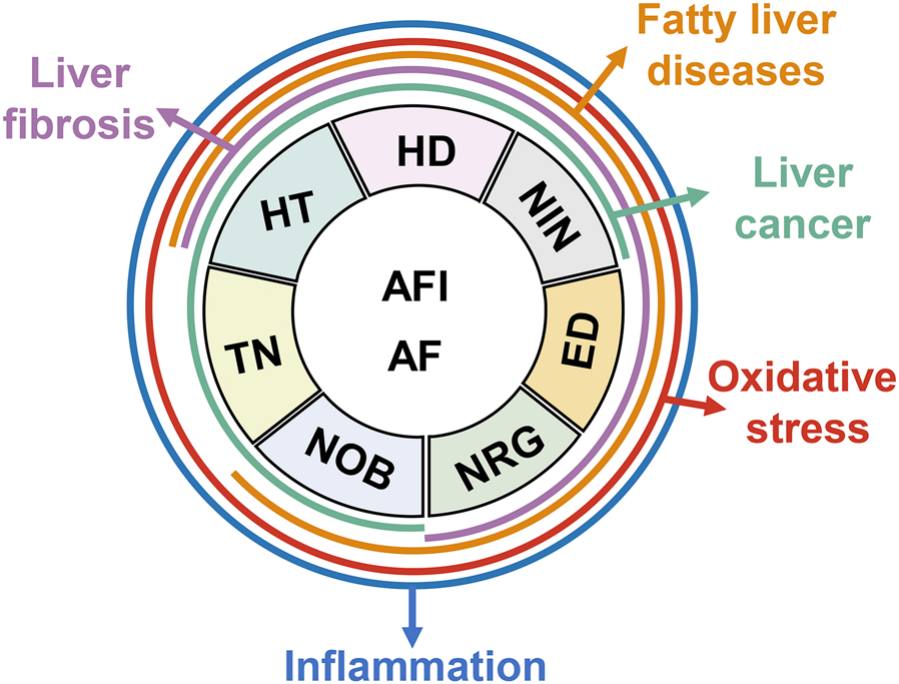
Pharmacological effects of flavonoids in AFI and AF and related liver diseases

In summary, even after taking these obstacles and concerns into account, the flavonoids in AFI and AF, including HD, NIN, NOB, NRG, TN, HT and ED, are valuable drug candidates for the treatment of various liver diseases that have attracted extensive attention for their significant anti-oxidative stress, anti-inflammatory, anti-fibrosis anti-lipotoxicity, and anti-cancer effects (Fig. 6). Therefore, there is an urgent need to investigate their pharmacokinetic properties and establish the dose-time-pharmacology-toxicology relationships of flavonoids isolated from AFI and AF and their derivatives. Further studies on the liver-specific targets and molecular mechanisms of HD and HT bioactivities and other ingredients isolated from AFI and AF are yet to be performed. These studies are of great significance not only for AFI- and AF-based drug discovery and development but also for the clinical application and improvement of AFI- and AF-containing formulations as therapeutic agents for the treatment of liver diseases.

## Data Availability

All the data used to support the findings of this study are available from the corresponding author upon reasonable request.

## Abbreviations

AFI: *Aurantii Fructus Immaturus*
AMPK: AMP-activated protein kinase
CCl_4_: Carbon tetrachloride
COX-2: Cyclooxygenase-2
ED: Eriodictyol
FAS: Fatty acid synthase
GPx: Glutathione peroxidase
HD: Hesperidin
HO-1: HEME oxygenase 1
HT: Hesperetin
HSC: Hepatic stellate cell
IL-6: Interleukin-6
iNOS: Inducible nitric oxide synthase
MMP: Mitochondrial membrane potential
NIN: Naringenin
NRG: Naringin
NOB: Nobiletin
NF-κB: Nuclear factor-kappa B
Nrf2: Nuclear factor erythroid 2-related factor 2
SOD: Superoxide dismutase
TGF-β1: Transforming growth factor-β1
TN: Tangeretin
TNF-α: Tumor necrosis factor-α

## References

[1] Li X, Liu R, Zhang L, Jiang Z (2017). The emerging role of AMP-activated protein kinase in cholestatic liver diseases. Pharmacol Res.

[2] Zhang Q, Ye M (2009). Chemical analysis of the Chinese herbal medicine Gan-Cao (licorice). J Chromatogr A.

[3] Tsai FJ, Liu X, Chen CJ, Li TM, Chiou JS, Chuang PH, Ko CH, Lin TH, Liao CC, Huang SM (2019). Chinese herbal medicine therapy and the risk of overall mortality for patients with liver cancer who underwent surgical resection in Taiwan. Complement Ther Med..

[4] Chen M, Men L, Wu H, Zhong G, Ou L, Li T, Guo Y, Lin H, Zhang J, Wang D (2019). A systematic review of the effectiveness and safety of Chinese herbal medicine formula Gualou Xiebai Banxia (GLXBBX) decoction for the treatment of stable angina pectoris. Medicine (Baltimore)..

[5] El-Sisi AEE, Sokar SS, Shebl AM, Mohamed DZ (2017). Antifibrotic effect of diethylcarbamazine combined with hesperidin against ethanol induced liver fibrosis in rats. Biomed Pharmacother.

[6] Li P, Zeng SL, Duan L, Ma XD, Dou LL, Wang LJ, Li P, Bi ZM, Liu EH (2016). Comparison of Aurantii Fructus Immaturus and Aurantii Fructus based on multiple chromatographic analysis and chemometrics methods. J Chromatogr A.

[7] Deshmukh NS, Stohs SJ, Magar CC, Kadam SB (2017). Citrus aurantium (bitter orange) extract: safety assessment by acute and 14-day oral toxicity studies in rats and the Ames Test for mutagenicity. Regul Toxicol Pharmacol.

[8] Feng S, Suh JH, Gmitter FG, Wang Y (2018). Differentiation between flavors of sweet orange (*Citrus sinensis*) and mandarin (*Citrus reticulata*). J Agric Food Chem.

[9] Favela-Hernandez JM, Gonzalez-Santiago O, Ramirez-Cabrera MA, Esquivel-Ferrino PC, Camacho-Corona Mdel R (2016). Chemistry and pharmacology of *Citrus sinensis*. Molecules.

[10] Huang W, Xiong ZH, Huang X, Chen X, Liu WP, Wang Y, Ren P (2012). Simultaneous UPLC analysis of three major flavonoids in granule decoctions of *Fructus aurantii*-type formulae. Pharmazie..

[11] Hosseini A, Sadeghnia HR, Rajabian A (2016). Protective effects of peel and seed extracts of *Citrus aurantium* on glutamate-induced cytotoxicity in PC12 cell line. Folia Neuropathol.

[12] Bai Y, Zheng Y, Pang W, Peng W, Wu H, Yao H, Li P, Deng W, Cheng J, Su W (2018). Identification and comparison of constituents of Aurantii Fructus and Aurantii Fructus Immaturus by UFLC-DAD-triple TOF-MS/MS. Molecules..

[13] Benavente-Garcia O, Castillo J (2008). Update on uses and properties of citrus flavonoids: new findings in anticancer, cardiovascular, and anti-inflammatory activity. J Agric Food Chem.

[14] Barreca D, Gattuso G, Bellocco E, Calderaro A, Trombetta D, Smeriglio A, Lagana G, Daglia M, Meneghini S, Nabavi SM (2017). Flavanones: citrus phytochemical with health-promoting properties. BioFactors.

[15] Li L, Zhang S, Xin Y, Sun J, Xie F, Yang L, Chen Z, Chen H, Liu F, Xuan Y (2018). Role of Quzhou Fructus Aurantii extract in preventing and treating acute lung injury and inflammation. Sci Rep..

[16] Bai YF, Wang SW, Wang XX, Weng YY, Fan XY, Sheng H, Zhu XT, Lou LJ, Zhang F (2019). The flavonoid-rich Quzhou Fructus Aurantii extract modulates gut microbiota and prevents obesity in high-fat diet-fed mice. Nutr Diabetes..

[17] Shu Y, He D, Li W, Wang M, Zhao S, Liu L, Cao Z, Liu R, Huang Y, Li H, et al. Hepatoprotective effect of *Citrus aurantium* L. against APAP-induced liver injury by regulating liver lipid metabolism and apoptosis. Int J Biol Sci. 2020; 16(5): 752-765. 10.7150/ijbs.40612 PMC7019131 32071546

[18] Duan L, Guo L, Liu K, Liu EH, Li P (2014). Characterization and classification of seven citrus herbs by liquid chromatography-quadrupole time-of-flight mass spectrometry and genetic algorithm optimized support vector machines. J Chromatogr A.

[19] Senchina DS, Hallam JE, Kohut ML, Nguyen NA, Perera MA (2014). Alkaloids and athlete immune function: caffeine, theophylline, gingerol, ephedrine, and their congeners. Exerc Immunol Rev..

[20] Kulkarni MV, Kulkarni GM, Lin CH, Sun CM (2006). Recent advances in coumarins and 1-azacoumarins as versatile biodynamic agents. Curr Med Chem.

[21] Yang C, Zhang L, Cao G, Feng J, Yue M, Xu Y, Dai B, Han Q, Guo X (2019). Effects of dietary supplementation with essential oils and organic acids on the growth performance, immune system, fecal volatile fatty acids, and microflora community in weaned piglets. J Anim Sci.

[22] Zhao S, Liu Z, Wang M, He D, Liu L, Shu Y, Song Z, Li H, Liu Y, Lu A (2018). Anti-inflammatory effects of Zhishi and Zhiqiao revealed by network pharmacology integrated with molecular mechanism and metabolomics studies. Phytomedicine.

[23] Chuang CC, Wen WC, Sheu SJ (2007). Origin identification on the commercial samples of Aurantii Fructus. J Sep Sci.

[24] He YJ, Zhu M, Zhou Y, Zhao KH, Zhou JL, Qi ZH, Zhu YY, Wang ZJ, Xie TZ, Tang Q, et al. Comparative investigation of phytochemicals among ten citrus herbs by ultra high performance liquid chromatography coupled with electrospray ionization quadrupole time-of-flight mass spectrometry and evaluation of their antioxidant properties. J Sep Sci. 2020. 10.1002/jssc.202000335 32506783

[25] Zhu J, Yu K, Chen X, Hu Z (2007). Comparison of two sample preconcentration strategies for the sensitivity enhancement of flavonoids found in Chinese herbal medicine in micellar electrokinetic chromatography with UV detection. J Chromatogr A.

[26] Okada N, Murakami A, Urushizaki S, Matsuda M, Kawazoe K, Ishizawa K (2017). Extracts of immature orange (*Aurantii fructus* immaturus) and citrus unshiu peel (*Citri unshiu* pericarpium) induce P-Glycoprotein and cytochrome P450 3A4 expression via upregulation of pregnane X receptor. Front Pharmacol..

[27] Wu Y, Zhang W, Li M, Cao D, Yang X, Gong J (2017). Nobiletin ameliorates ischemia-reperfusion injury by suppressing the function of Kupffer cells after liver transplantation in rats. Biomed Pharmacother.

[28] Hu L, Li L, Xu D, Xia X, Pi R, Xu D, Wang W, Du H, Song E, Song Y (2014). Protective effects of neohesperidin dihydrochalcone against carbon tetrachloride-induced oxidative damage in vivo and in vitro. Chem Biol Interact.

[29] Dusabimana T, Kim SR, Kim HJ, Park SW, Kim H (2019). Nobiletin ameliorates hepatic ischemia and reperfusion injury through the activation of SIRT-1/FOXO3a-mediated autophagy and mitochondrial biogenesis. Exp Mol Med.

[30] Shi Q, Song X, Fu J, Su C, Xia X, Song E, Song Y (2015). Artificial sweetener neohesperidin dihydrochalcone showed antioxidative, anti-inflammatory and anti-apoptosis effects against paraquat-induced liver injury in mice. Int Immunopharmacol.

[31] Khan MK, Rakotomanomana N, Loonis M, Dangles O (2010). Chemical synthesis of citrus flavanone glucuronides. J Agric Food Chem.

[32] Li C, Schluesener H (2017). Health-promoting effects of the citrus flavanone hesperidin. Crit Rev Food Sci Nutr.

[33] Parhiz H, Roohbakhsh A, Soltani F, Rezaee R, Iranshahi M (2015). Antioxidant and anti-inflammatory properties of the citrus flavonoids hesperidin and hesperetin: an updated review of their molecular mechanisms and experimental models. Phytother Res..

[34] Koyama Y, Brenner DA (2017). Liver inflammation and fibrosis. J Clin Invest..

[35] de Andrade KQ, Moura FA, dos Santos JM, de Araujo OR, de Farias Santos JC, Goulart MO (2015). Oxidative stress and inflammation in hepatic diseases: therapeutic possibilities of *N*-acetylcysteine. Int J Mol Sci.

[36] Chen M, Gu H, Ye Y, Lin B, Sun L, Deng W, Zhang J, Liu J (2010). Protective effects of hesperidin against oxidative stress of tert-butyl hydroperoxide in human hepatocytes. Food Chem Toxicol.

[37] Elhelaly AE, AlBasher G, Alfarraj S, Almeer R, Bahbah EI, Fouda MMA, Bungau SG, Aleya L, Abdel-Daim MM (2019). Protective effects of hesperidin and diosmin against acrylamide-induced liver, kidney, and brain oxidative damage in rats. Environ Sci Pollut Res Int.

[38] Li G, Chen MJ, Wang C, Nie H, Huang WJ, Yuan TD, Sun T, Shu KG, Wang CF, Gong Q (2014). Protective effects of hesperidin on concanavalin A-induced hepatic injury in mice. Int Immunopharmacol.

[39] Pari L, Karthikeyan A, Karthika P, Rathinam A (2015). Protective effects of hesperidin on oxidative stress, dyslipidaemia and histological changes in iron-induced hepatic and renal toxicity in rats. Toxicol Rep..

[40] Ansar S, Abudawood M, Alaraj ASA, Hamed SS (2018). Hesperidin alleviates zinc oxide nanoparticle induced hepatotoxicity and oxidative stress. BMC Pharmacol Toxicol..

[41] Hamdy SM, Sayed ON, Abdel Latif AKM, Abdel-Aziz AM, Amin AM (2016). Hesperidin and tiger nut reduced carcinogenicity of DMBA in female rats. Biomed Pharmacother.

[42] Al-Rikabi R, Al-Shmgani H, Dewir YH, El-Hendawy S. In vivo and in vitro evaluation of the protective effects of hesperidin in lipopolysaccharide-induced inflammation and cytotoxicity of cell. Molecules. 2020; 25:3. 10.3390/molecules25030478 PMC7038000 31979178

[43] Sajadimajd S, Khazaei M (2018). Oxidative stress and cancer: the role of Nrf2. Curr Cancer Drug Targets.

[44] Wardyn JD, Ponsford AH, Sanderson CM (2015). Dissecting molecular cross-talk between Nrf2 and NF-kappaB response pathways. Biochem Soc Trans.

[45] Kaur S, Nag A, Singh AK, Sharma K (2018). PPARgamma-targeting Potential for Radioprotection. Curr Drug Targets.

[46] Mahmoud AM (2014). Hesperidin protects against cyclophosphamide-induced hepatotoxicity by upregulation of PPARgamma and abrogation of oxidative stress and inflammation. Can J Physiol Pharmacol.

[47] Uchida K (2017). HNE as an inducer of COX-2. Free Radic Biol Med..

[48] Marx SG, Wentz MJ, Mackay LB, Schlembach D, Maul H, Fittkow C, Given R, Vedernikov Y, Saade GR, Garfield RE (2006). Effects of progesterone on iNOS, COX-2, and collagen expression in the cervix. J Histochem Cytochem.

[49] Su C, Xia X, Shi Q, Song X, Fu J, Xiao C, Chen H, Lu B, Sun Z, Wu S (2015). Neohesperidin dihydrochalcone versus CCl(4)-induced hepatic injury through different mechanisms: the implication of free radical scavenging and Nrf2 activation. J Agric Food Chem.

[50] Wang X, Hasegawa J, Kitamura Y, Wang Z, Matsuda A, Shinoda W, Miura N, Kimura K (2011). Effects of hesperidin on the progression of hypercholesterolemia and fatty liver induced by high-cholesterol diet in rats. J Pharmacol Sci..

[51] Leray V, Freuchet B, Le Bloc’h J, Jeusette I, Torre C, Nguyen P (2011). Effect of citrus polyphenol- and curcumin-supplemented diet on inflammatory state in obese cats. Br J Nutr.

[52] Sun YZ, Chen JF, Shen LM, Zhou J, Wang CF (2017). Anti-atherosclerotic effect of hesperidin in LDLr(−/−) mice and its possible mechanism. Eur J Pharmacol.

[53] Ding RB, Bao J, Deng CX (2017). Emerging roles of SIRT1 in fatty liver diseases. Int J Biol Sci..

[54] Iskender H, Dokumacioglu E, Sen TM, Ince I, Kanbay Y, Saral S (2017). The effect of hesperidin and quercetin on oxidative stress, NF-kappaB and SIRT1 levels in a STZ-induced experimental diabetes model. Biomed Pharmacother.

[55] Shimano H, Sato R (2017). SREBP-regulated lipid metabolism: convergent physiology—divergent pathophysiology. Nat Rev Endocrinol..

[56] Zhou Z, Zhong W, Lin H, Huang P, Ma N, Zhang Y, Zhou C, Lai Y, Huang S, Huang S (2017). Hesperidin protects against acute alcoholic injury through improving lipid metabolism and cell damage in zebrafish larvae. Evid Based Complement Alternat Med..

[57] Ohara T, Muroyama K, Yamamoto Y, Murosaki S (2015). A combination of glucosyl hesperidin and caffeine exhibits an anti-obesity effect by inhibition of hepatic lipogenesis in mice. Phytother Res..

[58] Schuppan D (2015). Liver fibrosis: common mechanisms and antifibrotic therapies. Clin Res Hepatol Gastroenterol..

[59] Elshazly SM, Mahmoud AA (2014). Antifibrotic activity of hesperidin against dimethylnitrosamine-induced liver fibrosis in rats. Naunyn Schmiedebergs Arch Pharmacol..

[60] Luo K. Signaling cross talk between TGF-beta/Smad and other signaling pathways. Cold Spring Harb Perspect Biol. 2017; 9:1. 10.1101/cshperspect.a022137 PMC5204325 27836834

[61] Cetin A, Ciftci O, Otlu A (2016). Protective effect of hesperidin on oxidative and histological liver damage following carbon tetrachloride administration in Wistar rats. Arch Med Sci..

[62] Perez-Vargas JE, Zarco N, Shibayama M, Segovia J, Tsutsumi V, Muriel P (2014). Hesperidin prevents liver fibrosis in rats by decreasing the expression of nuclear factor-kappaB, transforming growth factor-beta and connective tissue growth factor. Pharmacology.

[63] Wu FR, Jiang L, He XL, Zhu PL, Li J (2015). Effect of hesperidin on TGF-beta1/Smad signaling pathway in HSC. Zhongguo Zhong Yao Za Zhi..

[64] Abd-Elhakim YM, Ghoneim MH, Khairy MH, Eissa SA. Single or combined protective and therapeutic impact of taurine and hesperidin on carbon tetrachloride-induced acute hepatic injury in rat. Environ Sci Pollut Res Int. 2020. 10.1007/s11356-020-07895-1 32016862

[65] Morsy MA, Nair AB (2018). Prevention of rat liver fibrosis by selective targeting of hepatic stellate cells using hesperidin carriers. Int J Pharm.

[66] Lee KH, Yeh MH, Kao ST, Hung CM, Liu CJ, Huang YY, Yeh CC (2010). The inhibitory effect of hesperidin on tumor cell invasiveness occurs via suppression of activator protein 1 and nuclear factor-kappaB in human hepatocellular carcinoma cells. Toxicol Lett.

[67] Cheng EH, Levine B, Boise LH, Thompson CB, Hardwick JM (1996). Bax-independent inhibition of apoptosis by Bcl-XL. Nature.

[68] Banjerdpongchai R, Wudtiwai B, Khaw-On P, Rachakhom W, Duangnil N, Kongtawelert P (2016). Hesperidin from Citrus seed induces human hepatocellular carcinoma HepG2 cell apoptosis via both mitochondrial and death receptor pathways. Tumour Biol.

[69] Naz H, Tarique M, Ahamad S, Alajmi MF, Hussain A, Rehman MT, Luqman S, Hassan MI (2019). Hesperidin-CAMKIV interaction and its impact on cell proliferation and apoptosis in the human hepatic carcinoma and neuroblastoma cells. J Cell Biochem.

[70] Pez F, Lopez A, Kim M, Wands JR, Caron de Fromentel C, Merle P. Wnt signaling and hepatocarcinogenesis: molecular targets for the development of innovative anticancer drugs. J Hepatol. 2013; 59(5): 1107–17. 10.1016/j.jhep.2013.07.001 23835194

[71] Zaghloul RA, Elsherbiny NM, Kenawy HI, El-Karef A, Eissa LA, El-Shishtawy MM (2017). Hepatoprotective effect of hesperidin in hepatocellular carcinoma: involvement of Wnt signaling pathways. Life Sci.

[72] Fernandez-Bedmar Z, Anter J, Alonso-Moraga A, Martin de Las Mulas J, Millan-Ruiz Y, Guil-Luna S. Demethylating and anti-hepatocarcinogenic potential of hesperidin, a natural polyphenol of Citrus juices. Mol Carcinog. 2017; 56(6):1653–62. 10.1002/mc.22621 28130850

[73] Mahmoud AM, Mohammed HM, Khadrawy SM, Galaly SR (2017). Hesperidin protects against chemically induced hepatocarcinogenesis via modulation of Nrf2/ARE/HO-1, PPARgamma and TGF-beta1/Smad3 signaling, and amelioration of oxidative stress and inflammation. Chem Biol Interact.

[74] Mo’men YS, Hussein RM, Kandeil MA. Involvement of PI3K/Akt pathway in the protective effect of hesperidin against a chemically induced liver cancer in rats. J Biochem Mol Toxicol. 2019: e22305. 10.1002/jbt.22305 30779474

[75] Hasanin AH, Matboli M, Seleem HS (2020). Hesperidin suppressed hepatic precancerous lesions via modulation of exophagy in rats. J Cell Biochem.

[76] Joshi R, Kulkarni YA, Wairkar S (2018). Pharmacokinetic, pharmacodynamic and formulations aspects of Naringenin: an update. Life Sci.

[77] Chao X, Wang H, Jaeschke H, Ding WX (2018). Role and mechanisms of autophagy in acetaminophen-induced liver injury. Liver Int..

[78] Lv Y, Zhang B, Xing G, Wang F, Hu Z (2013). Protective effect of naringenin against acetaminophen-induced acute liver injury in metallothionein (MT)-null mice. Food Funct..

[79] Jayaraman J, Namasivayam N (2011). Naringenin modulates circulatory lipid peroxidation, anti-oxidant status and hepatic alcohol metabolizing enzymes in rats with ethanol induced liver injury. Fundam Clin Pharmacol.

[80] Hermenean A, Ardelean A, Stan M, Hadaruga N, Mihali CV, Costache M, Dinischiotu A (2014). Antioxidant and hepatoprotective effects of naringenin and its beta-cyclodextrin formulation in mice intoxicated with carbon tetrachloride: a comparative study. J Med Food.

[81] Malayeri A, Badparva R, Mombeini MA, Khorsandi L, Goudarzi M. Naringenin: a potential natural remedy against methotrexate-induced hepatotoxicity in rats. Drug Chem Toxicol. 2020:1–8. 10.1080/01480545.2020.1719132 31986916

[82] Renugadevi J, Prabu SM (2010). Cadmium-induced hepatotoxicity in rats and the protective effect of naringenin. Exp Toxicol Pathol.

[83] Jain A, Yadav A, Bozhkov AI, Padalko VI, Flora SJ (2011). Therapeutic efficacy of silymarin and naringenin in reducing arsenic-induced hepatic damage in young rats. Ecotoxicol Environ Saf.

[84] Esmaeili MA, Alilou M (2014). Naringenin attenuates CCl4 -induced hepatic inflammation by the activation of an Nrf2-mediated pathway in rats. Clin Exp Pharmacol Physiol.

[85] Chtourou Y, Fetoui H, Jemai R, Ben Slima A, Makni M, Gdoura R (2015). Naringenin reduces cholesterol-induced hepatic inflammation in rats by modulating matrix metalloproteinases-2,9 via inhibition of nuclear factor kappaB pathway. Eur J Pharmacol.

[86] Jayaraman J, Jesudoss VA, Menon VP, Namasivayam N (2012). Anti-inflammatory role of naringenin in rats with ethanol induced liver injury. Toxicol Mech Methods.

[87] Hamalainen M, Nieminen R, Vuorela P, Heinonen M, Moilanen E (2007). Anti-inflammatory effects of flavonoids: genistein, kaempferol, quercetin, and daidzein inhibit STAT-1 and NF-kappaB activations, whereas flavone, isorhamnetin, naringenin, and pelargonidin inhibit only NF-kappaB activation along with their inhibitory effect on iNOS expression and NO production in activated macrophages. Mediators Inflamm.

[88] Li S, Zhang Y, Sun Y, Zhang G, Bai J, Guo J, Su X, Du H, Cao X, Yang J (2019). Naringenin improves insulin sensitivity in gestational diabetes mellitus mice through AMPK. Nutr Diabetes..

[89] Allister EM, Mulvihill EE, Barrett PH, Edwards JY, Carter LP, Huff MW (2008). Inhibition of apoB secretion from HepG2 cells by insulin is amplified by naringenin, independent of the insulin receptor. J Lipid Res.

[90] Lin H, Zhou Z, Zhong W, Huang P, Ma N, Zhang Y, Zhou C, Lai Y, Huang S, An H (2017). Naringenin inhibits alcoholic injury by improving lipid metabolism and reducing apoptosis in zebrafish larvae. Oncol Rep.

[91] Wang Q, Ou Y, Hu G, Wen C, Yue S, Chen C, Xu L, Xie J, Dai H, Xiao H (2020). Naringenin attenuates non-alcoholic fatty liver disease by down-regulating the NLRP3/NF-kappaB pathway in mice. Br J Pharmacol.

[92] Hernandez-Aquino E, Muriel P (2018). Beneficial effects of naringenin in liver diseases: molecular mechanisms. World J Gastroenterol.

[93] Hernandez-Aquino E, Zarco N, Casas-Grajales S, Ramos-Tovar E, Flores-Beltran RE, Arauz J, Shibayama M, Favari L, Tsutsumi V, Segovia J (2017). Naringenin prevents experimental liver fibrosis by blocking TGFbeta-Smad3 and JNK-Smad3 pathways. World J Gastroenterol.

[94] Hernandez-Aquino E, Quezada-Ramirez MA, Silva-Olivares A, Casas-Grajales S, Ramos-Tovar E, Flores-Beltran RE, Segovia J, Shibayama M, Muriel P (2019). Naringenin attenuates the progression of liver fibrosis via inactivation of hepatic stellate cells and profibrogenic pathways. Eur J Pharmacol.

[95] Arul D, Subramanian P (2013). Naringenin (citrus flavonone) induces growth inhibition, cell cycle arrest and apoptosis in human hepatocellular carcinoma cells. Pathol Oncol Res..

[96] Ahmed OM, Ahmed AA, Fahim HI, Zaky MY. Quercetin and naringenin abate diethylnitrosamine/acetylaminofluorene-induced hepatocarcinogenesis in Wistar rats: the roles of oxidative stress, inflammation and cell apoptosis. Drug Chem Toxicol. 2019:1–12. 10.1080/01480545.2019.1683187 31665932

[97] Kang Q, Gong J, Wang M, Wang Q, Chen F, Cheng KW (2019). 6-C-(E-Phenylethenyl)Naringenin attenuates the stemness of hepatocellular carcinoma cells by suppressing Wnt/beta-catenin signaling. J Agric Food Chem.

[98] Kang Q, Gong J, Wang M, Wang Q, Chen F, Cheng KW (2020). Correction to 6-C-(E-Phenylethenyl)naringenin attenuates the stemness of hepatocellular carcinoma cells by suppressing Wnt/beta-catenin signaling. J Agric Food Chem.

[99] Yoshigai E, Machida T, Okuyama T, Mori M, Murase H, Yamanishi R, Okumura T, Ikeya Y, Nishino H, Nishizawa M (2013). Citrus nobiletin suppresses inducible nitric oxide synthase gene expression in interleukin-1beta-treated hepatocytes. Biochem Biophys Res Commun.

[100] He Z, Li X, Chen H, He K, Liu Y, Gong J, Gong J (2016). Nobiletin attenuates lipopolysaccharide/Dgalactosamineinduced liver injury in mice by activating the Nrf2 antioxidant pathway and subsequently inhibiting NFkappaBmediated cytokine production. Mol Med Rep..

[101] Guvenc M, Cellat M, Gokcek I, Ozkan H, Arkali G, Yakan A, Yurdagul Ozsoy S, Aksakal M (2020). Nobiletin attenuates acetaminophen-induced hepatorenal toxicity in rats. J Biochem Mol Toxicol.

[102] Li M, Zhao H, Wu J, Wang L, Wang J, Lv K, Liu S, Wang M, Guan W, Liu J (2020). Nobiletin protects against acute liver injury via targeting c-Jun N-terminal kinase (JNK)-induced apoptosis of hepatocytes. J Agric Food Chem.

[103] Yuk T, Kim Y, Yang J, Sung J, Jeong HS, Lee J (2018). Nobiletin inhibits hepatic lipogenesis via activation of AMP-activated protein kinase. Evid Based Complement Alternat Med..

[104] Qi G, Guo R, Tian H, Li L, Liu H, Mi Y, Liu X (2018). Nobiletin protects against insulin resistance and disorders of lipid metabolism by reprogramming of circadian clock in hepatocytes. Biochim Biophys Acta Mol Cell Biol Lipids..

[105] Kim YJ, Choi MS, Woo JT, Jeong MJ, Kim SR, Jung UJ. Long-term dietary supplementation with low-dose nobiletin ameliorates hepatic steatosis, insulin resistance, and inflammation without altering fat mass in diet-induced obesity. Mol Nutr Food Res. 2017; 61(8). 10.1002/mnfr.201600889 28116779

[106] Wei M, Shao Y, Liu QR, Wu QZ, Zhang X, Zhong MW, Liu SZ, Zhang GY, Hu SY (2018). Bile acid profiles within the enterohepatic circulation in a diabetic rat model after bariatric surgeries. Am J Physiol Gastrointest Liver Physiol.

[107] Satsu H, Awara S, Unno T, Shimizu M (2018). Suppressive effect of nobiletin and epicatechin gallate on fructose uptake in human intestinal epithelial Caco-2 cells. Biosci Biotechnol Biochem.

[108] Ohnishi H, Asamoto M, Tujimura K, Hokaiwado N, Takahashi S, Ogawa K, Kuribayashi M, Ogiso T, Okuyama H, Shirai T (2004). Inhibition of cell proliferation by nobiletin, a dietary phytochemical, associated with apoptosis and characteristic gene expression, but lack of effect on early rat hepatocarcinogenesis in vivo. Cancer Sci.

[109] Shi MD, Liao YC, Shih YW, Tsai LY (2013). Nobiletin attenuates metastasis via both ERK and PI3K/Akt pathways in HGF-treated liver cancer HepG2 cells. Phytomedicine.

[110] Adil M, Kandhare AD, Ghosh P, Venkata S, Raygude KS, Bodhankar SL (2016). Ameliorative effect of naringin in acetaminophen-induced hepatic and renal toxicity in laboratory rats: role of FXR and KIM-1. Ren Fail.

[111] Ahmed OM, Fahim HI, Ahmed HY, Al-Muzafar HM, Ahmed RR, Amin KA, El-Nahass ES, Abdelazeem WH (2019). The preventive effects and the mechanisms of action of navel orange peel hydroethanolic extract, naringin, and naringenin in *N*-acetyl-p-aminophenol-induced liver injury in wistar rats. Oxid Med Cell Longev..

[112] Dong D, Xu L, Yin L, Qi Y, Peng J (2015). Naringin prevents carbon tetrachloride-induced acute liver injury in mice. Journal of Functional Foods..

[113] Jagetia GC, Reddy TK (2011). Alleviation of iron induced oxidative stress by the grape fruit flavanone naringin in vitro. Chem Biol Interact.

[114] Caglayan C, Temel Y, Kandemir FM, Yildirim S, Kucukler S (2018). Naringin protects against cyclophosphamide-induced hepatotoxicity and nephrotoxicity through modulation of oxidative stress, inflammation, apoptosis, autophagy, and DNA damage. Environ Sci Pollut Res Int.

[115] Rathi VK, Das S, Parampalli Raghavendra A, Rao BSS. Naringin abates adverse effects of cadmium-mediated hepatotoxicity: an experimental study using HepG2 cells. J Biochem Mol Toxicol. 2017; 31:8. 10.1002/jbt.21915 28422390

[116] Maatouk M, Mustapha N, Mokdad-Bzeouich I, Chaaban H, Ioannou I, Ghedira K, Ghoul M, Chekir-Ghedira L (2018). Heated naringin mitigate the genotoxicity effect of Mitomycin C in BALB/c mice through enhancing the antioxidant status. Biomed Pharmacother.

[117] Lv Z, Wu W, Ge S, Jia R, Lin T, Yuan Y, Kuang H, Yang B, Wu L, Wei J (2018). Naringin protects against perfluorooctane sulfonate-induced liver injury by modulating NRF2 and NF-kappaB in mice. Int Immunopharmacol.

[118] Gelen V, Sengul E, Yildirim S, Atila G (2018). The protective effects of naringin against 5-fluorouracil-induced hepatotoxicity and nephrotoxicity in rats. Iran J Basic Med Sci..

[119] Rodriguez V, Plavnik L, Tolosa de Talamoni N. Naringin attenuates liver damage in streptozotocin-induced diabetic rats. Biomed Pharmacother. 2018; 105: 95-102. 10.1016/j.biopha.2018.05.120 29852394

[120] El-Desoky AH, Abdel-Rahman RF, Ahmed OK, El-Beltagi HS, Hattori M. Anti-inflammatory and antioxidant activities of naringin isolated from Carissa carandas L.: In vitro and in vivo evidence. Phytomedicine. 2018; 42:126–34. 10.1016/j.phymed.2018.03.051 29655678

[121] Pu P, Gao DM, Mohamed S, Chen J, Zhang J, Zhou XY, Zhou NJ, Xie J, Jiang H (2012). Naringin ameliorates metabolic syndrome by activating AMP-activated protein kinase in mice fed a high-fat diet. Arch Biochem Biophys.

[122] Adil M, Kandhare AD, Visnagri A, Bodhankar SL (2015). Naringin ameliorates sodium arsenite-induced renal and hepatic toxicity in rats: decisive role of KIM-1, Caspase-3, TGF-beta, and TNF-alpha. Ren Fail.

[123] Shi H, Shi H, Ren F, Chen D, Chen Y, Duan Z (2017). Naringin in Ganshuang Granule suppresses activation of hepatic stellate cells for anti-fibrosis effect by inhibition of mammalian target of rapamycin. J Cell Mol Med.

[124] El-Mihi KA, Kenawy HI, El-Karef A, Elsherbiny NM, Eissa LA (2017). Naringin attenuates thioacetamide-induced liver fibrosis in rats through modulation of the PI3K/Akt pathway. Life Sci.

[125] Banjerdpongchai R, Wudtiwai B, Khawon P (2016). Induction of human hepatocellular carcinoma HepG2 cell apoptosis by naringin. Asian Pac J Cancer Prev.

[126] Suguro R, Pang XC, Yuan ZW, Chen SY, Zhu YZ, Xie Y (2020). Combinational applicaton of silybin and tangeretin attenuates the progression of non-alcoholic steatohepatitis (NASH) in mice via modulating lipid metabolism. Pharmacol Res.

[127] Omar HA, Mohamed WR, Arab HH, Arafa el SA (2016). Tangeretin alleviates cisplatin-induced acute hepatic injury in rats: targeting MAPKs and apoptosis. PLoS ONE.

[128] Liang F, Fang Y, Cao W, Zhang Z, Pan S, Xu X (2018). Attenuation of tert-butyl hydroperoxide (t-BHP)-induced oxidative damage in HepG2 cells by tangeretin: relevance of the Nrf2-ARE and MAPK Signaling pathways. J Agric Food Chem.

[129] Li Y, Liu R, Wu J, Li X (2020). Self-eating: friend or foe? The emerging role of autophagy in fibrotic diseases. Theranostics..

[130] Zheng J, Shao Y, Jiang Y, Chen F, Liu S, Yu N, Zhang D, Liu X, Zou L (2019). Tangeretin inhibits hepatocellular carcinoma proliferation and migration by promoting autophagy-related BECLIN1. Cancer Manag Res..

[131] Duran Y,Karaboga I. Effect of hesperetin on systemic inflammation and hepatic injury after blunt chest trauma in rats. Biotech Histochem. 2019:1–8. 10.1080/10520295.2019.1691265 31850807

[132] Kong R, Wang N, Luo H, Lu J (2018). Hesperetin mitigates bile duct ligation-induced liver fibrosis by inhibiting extracellular matrix and cell apoptosis via the TGF-beta1/Smad pathway. Curr Mol Med.

[133] Wan J, Kuang G, Zhang L, Jiang R, Chen Y, He Z, Ye D (2020). Hesperetin attenuated acetaminophen-induced hepatotoxicity by inhibiting hepatocyte necrosis and apoptosis, oxidative stress and inflammatory response via upregulation of heme oxygenase-1 expression. Int Immunopharmacol.

[134] Chen X, Ding HW, Li HD, Huang HM, Li XF, Yang Y, Zhang YL, Pan XY, Huang C, Meng XM, et al. Hesperetin derivative-14 alleviates inflammation by activating PPAR-gamma in mice with CCl4-induced acute liver injury and LPS-treated RAW264.7 cells. Toxicol Lett. 2017; 274:51–63. 10.1016/j.toxlet.2017.04.008 28428136

[135] Li WX, Chen X, Yang Y, Huang HM, Li HD, Huang C, Meng XM, Li J (2017). Hesperitin derivative-11 suppress hepatic stellate cell activation and proliferation by targeting PTEN/AKT pathway. Toxicology.

[136] Meng HW, You HM, Yang Y, Zhang YL, Meng XM, Ma TT, Huang C, Li J (2019). 4-Methylcoumarin-[5,6-g]-hesperetin attenuates inflammatory responses in alcoholic hepatitis through PPAR-gamma activation. Toxicology.

[137] Zhu GF, Guo HJ, Huang Y, Wu CT, Zhang XF (2015). Eriodictyol, a plant flavonoid, attenuates LPS-induced acute lung injury through its antioxidative and anti-inflammatory activity. Exp Ther Med..

[138] Xie G, Meng X, Wang F, Bao Y, Huo J (2017). Eriodictyol attenuates arsenic trioxide-induced liver injury by activation of Nrf2. Oncotarget..

[139] Wang Z, Lan Y, Chen M, Wen C, Hu Y, Liu Z, Ye L (2017). Eriodictyol, not its glucuronide metabolites, attenuates acetaminophen-induced hepatotoxicity. Mol Pharm.

[140] Kwon EY,Choi MS. Dietary Eriodictyol Alleviates Adiposity, Hepatic Steatosis, Insulin Resistance, and Inflammation in Diet-Induced Obese Mice. Int J Mol Sci. 2019; 20:5. 10.3390/ijms20051227 PMC6429409 30862092

[141] Afridi R, Khan AU, Khalid S, Shal B, Rasheed H, Ullah MZ, Shehzad O, Kim YS, Khan S (2019). Anti-hyperalgesic properties of a flavanone derivative Poncirin in acute and chronic inflammatory pain models in mice. BMC Pharmacol Toxicol..

[142] Park HY, Ha SK, Eom H, Choi I (2013). Narirutin fraction from citrus peels attenuates alcoholic liver disease in mice. Food Chem Toxicol.

[143] Hu SW, Zhong KR, Yang JY, Su L, Jiang YY, Liu B (2018). Study on difference of flavonoids content in stems and leaves of Mentha Haplocalycis Herba in different harvest periods. Zhongguo Zhong Yao Za Zhi..

[144] Sansone F, Rossi A, Del Gaudio P, De Simone F, Aquino RP, Lauro MR (2009). Hesperidin gastroresistant microparticles by spray-drying: preparation, characterization, and dissolution profiles. AAPS PharmSciTech.

